# Neuroimaging of Pediatric Intracerebral Hemorrhage

**DOI:** 10.3390/jcm9051518

**Published:** 2020-05-18

**Authors:** Peter B. Sporns, Marios-Nikos Psychogios, Heather J. Fullerton, Sarah Lee, Olivier Naggara, Grégoire Boulouis

**Affiliations:** 1 Department of Neuroradiology, Clinic for Radiology & Nuclear Medicine, University Hospital Basel, 4031 Basel, Switzerland; marios.psychogios@usb.ch; 2 Department of Diagnostic and Interventional Neuroradiology, University Medical Center Hamburg-Eppendorf, 20246 Hamburg, Germany; 3 Department of Neurology and Pediatrics, University of California, San Francisco, CA 94143, USA; heather.fullerton@ucsf.edu; 4 Division of Child Neurology, Department of Neurology, Stanford University, Palo Alto, CA 94305, USA; slee10@stanford.edu; 5 Pediatric Radiology Department, Necker Enfants Malades & GHU Paris, Sainte-Anne Hospital, Institut de Psychiatrie et Neurosciences de Paris (IPNP), UMR S1266, INSERM, Université de Paris, 75015 Paris, France; o.naggara@ghu-paris.fr (O.N.); gregoireboulouis@gmail.com (G.B.)

**Keywords:** neuroimaging, intracerebral haemorrhage, paediatric stroke

## Abstract

Hemorrhagic strokes account for half of all strokes seen in children, and the etiologies of these hemorrhagic strokes differ greatly from those seen in adult patients. This review gives an overview about incidence and etiologies as well as presentation of children with intracerebral hemorrhage and with differential diagnoses in the emergency department. Most importantly it describes how neuroimaging of children with intracerebral hemorrhage should be tailored to specific situations and clinical contexts and recommends specific imaging protocols for acute and repeat imaging. In this context it is important to keep in mind the high prevalence of underlying vascular lesions and adapt the imaging protocol accordingly, meaning that vascular imaging plays a key role regardless of modality. Magnetic resonance imaging (MRI), including advanced sequences, should be favored whenever possible at the acute phase.

## 1. Introduction

While accounting for a minority of adult strokes, hemorrhagic strokes account for half of all strokes seen in children, and occur in approximately 1–2 per 100,000 children per year [1,2]. The etiologies of hemorrhagic stroke differ in children as well; hypertensive hemorrhage rarely occurs in children, and cerebral amyloid angiopathy is almost exclusively an adult disease [3]. Trauma is the most common cause of intracerebral hemorrhage (ICH) in children, and the distinction between traumatic versus spontaneous hemorrhage can be challenging. Spontaneous ICH in children is most often caused by an underlying structural lesion, most often a congenital vascular malformation. Patterns of hemorrhage can provide a clue to etiology—a child with an ICH extending into the ventricles or subarachnoid space most likely has a brain arteriovenous malformation (AVM), a child with a pure ICH (involving the parenchyma only) has about an equal chance of an AVM or cavernous malformation [4]. Cerebral aneurysms rarely cause intraparenchymal hemorrhage, but are the most common cause of pure subarachnoid hemorrhage, even in children. Arteriovenous fistulas (AVF) in children, compared to adults, are more likely pial (as opposed to dural) and congenital (as opposed to acquired) [5]. While most AVMs lesions in children are spontaneous, features suggesting an underlying genetic syndrome include multiple AVMs, AVMs in unusual locations (e.g., spinal), and vascular birthmarks, in addition to a positive family history (autosomal dominant inheritance pattern) [6]. Multiple cavernous malformations similarly suggest a genetic syndrome, and vascular birthmarks can again serve as a clue [7] 

In this review, we therefore give an overview about the clinical presentation of pediatric patients with intracerebral hemorrhage and with differential diagnoses in the emergency department. We also describe how neuroimaging of children with intracerebral hemorrhage should be tailored to specific situations and clinical contexts and recommend specific imaging protocols for acute and repeat imaging.

## 2. Diagnosis of Intracerebral Hemorrhage in the Emergency Department

Presenting symptoms of ICH in children can be nonspecific, particularly without preceding head trauma to raise initial suspicion. Headache is the most common feature of ICH in non-neonates, occurring in up to 80% of children [8,9,10]. While acute ‘thunderclap headache’ is often associated with aneurysmal subarachnoid hemorrhage in adults, the quality and severity of the headache in children may be more subtle, and difficult to distinguish from migraine [9]. Generalized or focal seizures occur in 20%–40% [11,12,13], focal neurologic deficits in 13%–50% [9,10,14], and signs of increased intracranial pressure (nausea, vomiting, depressed level of consciousness) in 50%–60% of children [10,11,15]. Older children (≥6 years) are more likely to present with focal neurologic deficits and are also more likely to be able to articulate the quality and severity of headache, prompting emergent work-up. Younger children, on the other hand, more often present with non-localizing symptoms such as irritability, difficulty feeding, and lethargy, making it difficult to decide whom to image emergently [9,14]. Mackay, et al. investigated features distinguishing true stroke from stroke mimics in the emergency department, and found that acute onset of symptoms, vomiting, altered mental status, inability to walk, abnormal Glasgow coma scale score (GCS) and coma were associated with hemorrhagic stroke on univariate analysis; importantly, seizure at stroke onset did not predict a stroke mimic, highlighting a key difference between stroke presentation in children versus adults [16]. Initial assessment in the emergency department should be rapid, and focus on signs of increased ICP, such as pupillary size and response (fixed, dilated or sluggish pupils or anisocoria); eye movements (cranial nerve VI palsies or forced downgaze), and optic discs when possible (papilledema). Level of consciousness may be evaluated with the Glasgow Coma Scale and focal neurologic deficits with a NIH stroke scale when possible [17]. Neurocutaneous or other genetic stigmata may also raise suspicion for ICH in a child with otherwise nonspecific symptoms. For any child acutely deteriorating, stabilizing airway, breathing and circulation is critical prior to emergent neuroimaging.

## 3. Acute Imaging Work-up

The choice of neuroimaging modality should always be adjusted to clinical presentation and suspected cause. In children with acute focal neurological deficits, most pediatric stroke guidelines recommend brain magnetic resonance imaging (MRI) as first imaging modality especially in suspected ischemic stroke, where computed tomography (CT) may be less sensitive for detection of early ischemic changes [17,18]. Yet, in children with suspected intracranial hemorrhage, CT has several advantages, including greater accessibility and faster acquisition time [3] limiting the risk of requiring sedation for imaging acquisition, hence often counterbalancing the disadvantages of radiation and iodine contrast exposure [17], in the context of a life-threatening condition. Altogether, MRI should be preferred in stable and calm children, and CT favored in children with vigilance alteration, agitation or acutely worsening neurological symptoms.

After the positive diagnosis, careful evaluation of risk factors for secondary neurological deterioration is mandated. Pediatric counterparts to the adult ICH scoring system [19] have been developed in order to predict severity and outcome of pediatric ICH. These scores consider a number of factors, including ICH/brain volume ratio; infratentorial location; intraventricular hemorrhage; hydrocephalus; mass effect/brain herniation; and altered mental status [10,20]. Higher ICH score has been associated with worse outcomes, and presence of markers of severity should prompt consideration for immediate transfer to a pediatric neuro-intensive care environment.

**Figure 1:**
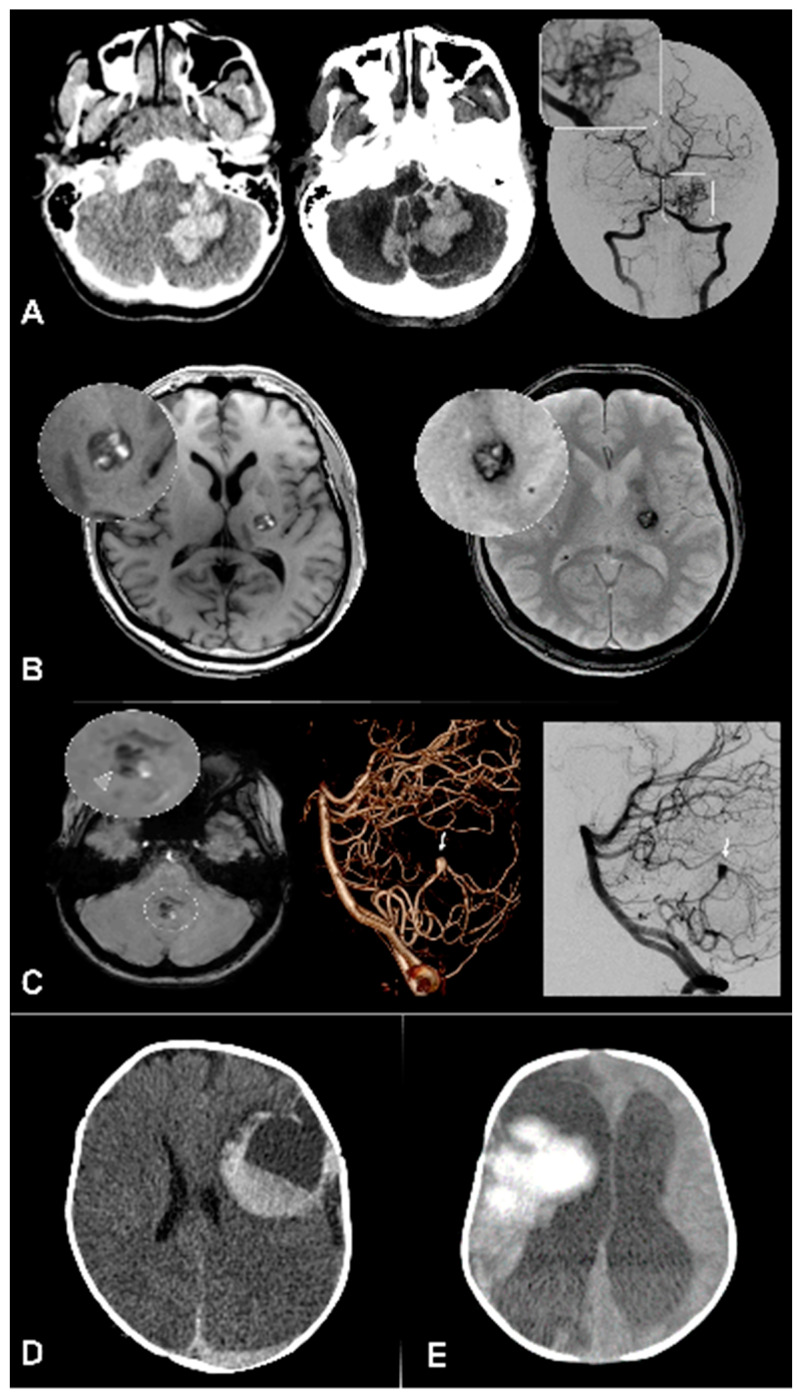
Pediatric intracerebral hemorrhage (ICH) main etiologies. (**A**) Left cerebellar mid peduncular hemorrhage, demonstrated on computed tomography (CT) and CT-angiography; digital subtraction angiography (DSA) (right and inset) shows malformative vessels with PICA and AICA feeders, a nidus and deep venous outflow corresponding to an arteriovenous malformation. (**B**) Left internal capsule cavernoma, with distinctive heterogeneous signal on T1 (left) and hyposignal on T2 * (right). (**C**) Smaller ICH in the cerebellar vermis (arrowhead). DSA shows a fusiform, likely dissecting, aneurysm. (**D**) Clotting factor deficiency revealed by a left supratentorial bleed. The presence of a fluid level is suggestive of a coagulation deficit. (**E**) Hemorrhagic transformation of a large right hemispheric ischemic stroke.

Intracranial vascular imaging (CT or MR angiography) should then systematically be acquired as part of the initial diagnostic examination. Indeed, there is a high prevalence of readily identifiable vascular lesions such as arteriovenous malformations (AVM), arteriovenous fistulas (AVF), or aneurysms in children with ICH. (Figure 1) If identified, high recurrence risk sectors (ectasia or arterial aneurysm) should be searched for so as to guide therapeutic management, and prevent early recurrent bleeding [17]. 

**Figure 2:**
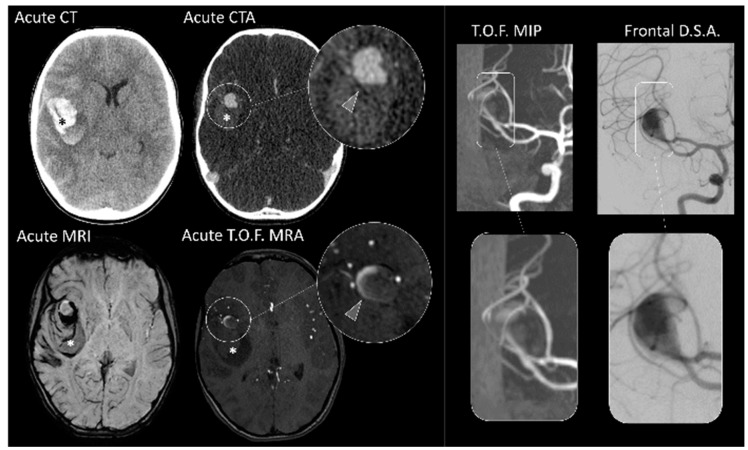
Vascular imaging in pediatric ICH. Admission non-contrast CT with intracerebral hemorrhage (asterisk). Corresponding CTA shows evidence of large aneurysm of the right MCA. In the MRI (bottom row and right panel)) of the same patient, TOF-angiography does not clearly show the aneurysm. The DSA clearly visualizes aneurysm morphology.

Digital subtraction angiography (DSA) has a central role for further characterization of vascular anomalies, and to investigate ICHs with undetermined etiology, due to its higher spatial and temporal resolutions and consequent higher sensitivity for intracranial shunts detection. (Figure 1 and Figure 2) The optimal timing for DSA realization is unknown, but should theoretically be performed as soon as possible in children with ICH of unknown origin to identify and treat occult vascular malformations. Of note, DSA is a low risk and often high yield examination in children: a recent analysis of pediatric patients revealed a 0% complication rate during the procedure and a 0.4% postprocedural complication rate [21,22]. 

In the absence of intracranial arteriovenous shunt, the second most prevalent etiology are cavernomas, more conspicuously detected using MRI. Similarly, hemorrhagic brain tumors should be searched for using contrast enhanced MRI [3]. 

To summarize, the optimal acute imaging sequence for a child with ICH would be CT or MR with vascular imaging (CTA-MRA), followed with gadolinium enhanced MRI if the initial modality was CT, finally completed DSA in the absence of identifiable cause.

## 4. Advanced Imaging and MR Protocol

The specificity of ICH etiological spectrum in children makes advanced MR imaging techniques particularly useful for the routine evaluation of pediatric ICH etiology.

**Figure 3:**
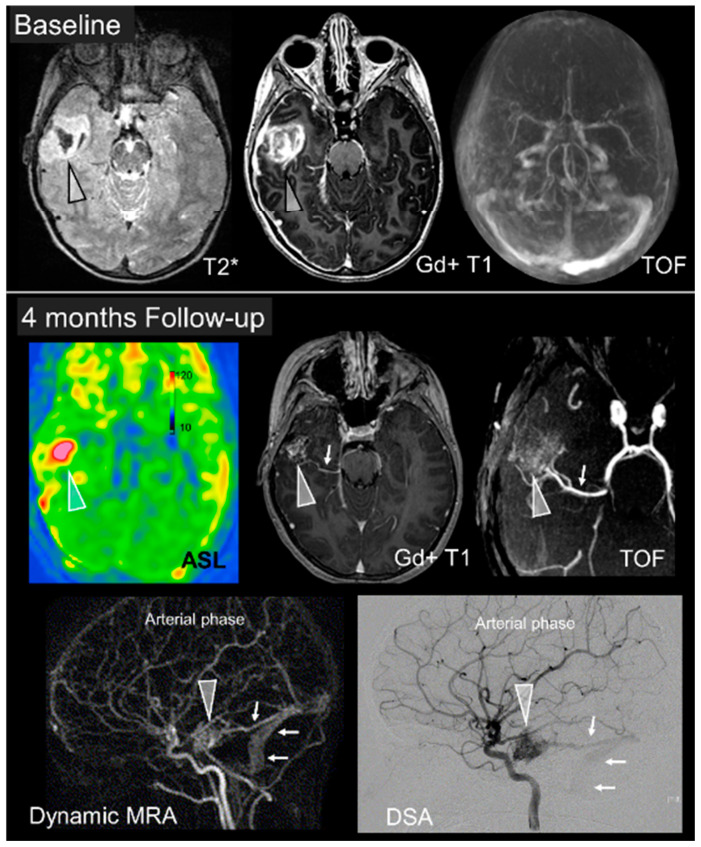
Advanced imaging. MRI images of a 5-year-old girl presenting with acute headaches and confusion. The top row shows an early subacute intracerebral hemorrhage (Arrowhead, HypotT2* and HyperT1) with no detectable vascular anomaly on the TOF sequence. DSA was also found to be normal at that time. The bottom panels show the imaging appearance 4 months thereafter; with increased cerebral blood flow (CBF) within a vascular nidus (Arrowhead) seen on the ASL sequence, post contrast T1, and TOF. Dynamic MRA shows early enhancement of cerebral veins and a nidus within the parenchyma characterizing a brain arteriovenous malformation (AVM), confirmed on the DSA acquired the next day.

As arteriovenous shunts are the most prominent cause for hemorrhage in children, perfusion imaging such as arterial spin labelling, and dynamic vascular imaging such as time-resolved multiphase MR-angiography can give immediate insight on the underlying etiology and help tailor therapeutic management (See Figure 3—Advanced imaging)

Arterial spin labelling is a MR based non-contrast perfusion technique that has been shown to identify areas of high cerebral blood flow in pediatric AVMs with good sensitivity [23]. Quantifying its added value in the routine acute evaluation of pediatric ICH is an unmet need, but it is a useful and easy-to-use adjunct in clinical practice.

Similarly, dynamic MR-angiography can provide key information with regards to the presence of an arteriovenous shunt, as well as an accurate preliminary delineation of arterial feeders, nidus sectors, venous outflow and their respective angiodynamics.

Altogether, when acquiring MRI for the etiological work-up of a child with pediatric ICH, the following sequence protocol is advisable:

T2* Gradient Echo, or its 3D susceptibility weighted imaging derivatives; for the positive the diagnosis of blood breakdown products and the identification of other lesions (e.g., distinct cavernomas in cavernomatosis), to detect intraventricular blood.

Diffusion weighted imaging, to identify areas of restricted diffusion that can point towards a hemorrhagic transformation of an acute ischemic stroke or towards a venous infarction in the context of cerebral venous thrombosis.

FLAIR or T2 (in children younger than 2 years old), to identify pre-existing brain lesions, including brain tumors, and quantify edema, to evaluate ventricles volume and hydrocephalus.

Pre contrast T1.

Whole brain time-of-flight angiography, to identify macro-vascular lesions.

Arterial spin labeling (ASL) perfusion, to identify arteriovenous shunts.

Dynamic MR-angiography, to identify and characterize high flow arteriovenous shunts.

Post-contrast T1, to identify enhancing tumors, delineate an arteriovenous malformation or other vascular malformations.

## 5. Specific Considerations According to Suspected Cause

### 5.1. Arteriovenous Malformation

In most vascular pathologies (AVMs, AVFs, and aneurysms) except cavernous malformation (CM), DSA will be key for further lesion characterization but a CM may also be accompanied by a second vascular lesion, such as an AVM, in which case a DSA may be useful. The major differential after diagnosis of ICH in children is the presence of an AVM as it is the most common vascular lesion in this age group, with a hemorrhage risk of up to 6% per year and a mortality rate of up to 25% per hemorrhage [24,25,26]. Treatment decisions for AVM greatly rely on DSA because it offers the greatest resolution of the nidus region and feeding arteries. Around 15% of cerebral AVMs receive some blood supply from meningeal arteries that are usually not visible on MRA [27,28]. Moreover, DSA and especially 3D-DSA is superior to CTA/MRA for assessment of outflow stenosis and deep venous drainage [29]. Newer technologies (4D-DSA) combine the aforementioned exceptional spatial resolution of DSA with a sufficient temporal resolution further enhancing treatment decisions [30,31]. At baseline, parts of the AVM architecture may be obscured by fresh clots causing the need for reimaging after clot resorption [32,33], and prompting caution with regards to draining vein obstruction.

### 5.2. Cavernous Malformation

Cavernous malformations (CM) is amongst most frequent causes for non-traumatic pediatric ICH. It typically appears on T2 * Gradient Echo weighted sequences as a multilobulated well-defined lesion with blooming hypointensity [34,35]. The presence of distinct signal intensities on T1 within the lesions lobules is highly suggestive of the diagnosis. A genetic or post radiation pathogenesis may be present in case of multiple CMs [36]. Identifying a developmental venous anomaly (DVA) in the immediate vicinity of the lesion adds to the imaging suspicion, and can guide surgical approach.

### 5.3. Aneurysms and Pseudo-Aneurysms

CTA/MRA are often used as first screening for aneurysms [37,38]; however, DSA is still the gold standard with highest detection rates (97% of patients versus 80% of the time without DSA), especially for the detection of small aneurysms under 4 mm [11,39,40,41]. An important advantage of DSA is the possibility to perform rotational angiographies and thereby create 3D reconstructions of every aspect of the aneurysm. This allows for a highly detailed depiction of the aneurysm anatomy, enabling for best possible planning of therapeutic strategies [42]. Moreover, for example in anterior communicating aneurysms the contralateral ICA and A1 can be depicted and merged with the ipsilateral side to plan endovascular therapy [43]. 

## 6. Follow up and Repeat Imaging 

When the initial imaging work-up is inconclusive, and in the absence of systemic concurrent causes, repeated brain imaging should be considered as all structural underlying etiologies may be masked by the acute mass effect caused by the hemorrhage. Vascular lesions can be masked or partially destroyed by the initial hemorrhage and the risk of rebleeding is hypothesized to be higher when the clot retracts [12]. 

Some indirect arguments may point to the presence of an underlying parenchymal lesion. This most notably includes the early detection of peri-hematoma brain edema. Indeed, edema typically develops after 48–72 h, hence important edema at baseline suggest the presence of a pre-existing tumoral lesion, which should prompt early re-imaging so as not to delay etiological care.

The timing of repeat imaging should be tailored to specific clinical context and should include repeat DSA. Common temporal landmarks include after mass effect contraction (within three weeks), and after complete hemorrhage resorption (within three to six months) if intermediate control remained inconclusive.

## 7. Conclusions

Neuroimaging of children with intracerebral hemorrhage should be tailored to specific situations and clinical contexts. Yet, one must keep in mind the high prevalence of underlying vascular lesions and adapt imaging protocol accordingly. Vascular imaging plays a key role regardless of modality. MRI, including advanced sequences, should be favored whenever possible at the acute phase.
